# Targeting Surface Markers in Anaplastic Thyroid Cancer: Future Directions in Ligand-bound Therapy

**DOI:** 10.1210/jendso/bvaf035

**Published:** 2025-02-27

**Authors:** Janice Pakkianathan, Samuel Chan, Joseph Cruz, Kennedi Ewan, Alfred A Simental, Salma Khan

**Affiliations:** Division of Biochemistry, Center for Health Disparities & Molecular Medicine, Loma Linda University School of Medicine, Loma Linda, CA 92350, USA; Division of Biochemistry, Center for Health Disparities & Molecular Medicine, Loma Linda University School of Medicine, Loma Linda, CA 92350, USA; Division of Biochemistry, Center for Health Disparities & Molecular Medicine, Loma Linda University School of Medicine, Loma Linda, CA 92350, USA; Division of Biochemistry, Center for Health Disparities & Molecular Medicine, Loma Linda University School of Medicine, Loma Linda, CA 92350, USA; Otolaryngology, Loma Linda University School of Medicine, Loma Linda, CA 92350, USA; Division of Biochemistry, Center for Health Disparities & Molecular Medicine, Loma Linda University School of Medicine, Loma Linda, CA 92350, USA; Otolaryngology, Loma Linda University School of Medicine, Loma Linda, CA 92350, USA

**Keywords:** radioligand, surface marker, NIS, LAT-1, VDR, BNCT

## Abstract

Anaplastic thyroid cancer (ATC) is the rarest and most aggressive form of thyroid cancer, known for its highly variable nature and poor prognosis, primarily due to the lack of effective treatments. While conventional therapies have had limited success, there remains an urgent need for novel therapeutic approaches to combat this disease. ATC tumors are resistant to the standard radioiodine therapy because they lack the sodium/iodide symporter (NIS), which is necessary for iodine uptake. However, recent advances in theranostics targeting cell surface markers have opened new avenues for treating ATC. We used the PubMed database and Google search engine to identify relevant articles using combinations of specific keywords related to the topic of interest, focusing on each surface marker. This review explores multiple surface markers identified in ATC and their promising roles for delivering therapeutic agents into tumors, inducing cell death. Several promising markers, including prostate-specific membrane antigen, vitamin D receptor, IGF-1 receptor, programmed death-ligand 1, epidermal growth factor receptor, and L-type amino acid transporter 1 (LAT-1), have been found in ATC and could serve as effective targets for delivering therapeutic agents to tumors, inducing cell death. Restoring NIS expression is also explored as a potential therapy for ATC. Additionally, boron neutron capture therapy, which utilizes LAT-1 expression, is highlighted as a future therapeutic option due to its ability to selectively target tumor cells while minimizing damage to surrounding healthy tissue. These strategies offer the potential to overcome many of the challenges associated with ATC, improving patient outcomes and overall survival.

Thyroid cancer is the most common cancer associated with the endocrine system [1]. Histologically, thyroid cancer is classified into 5 groups: papillary and follicular (90%), medullary (5%), poorly differentiated (5%), and anaplastic (1%); the latter type is the rarest yet most aggressive and resistant to treatment [2]. Anaplastic thyroid carcinoma (ATC) makes up only 1% to 2% of all thyroid cancer cases and has a global incidence of only 1 to 2 people per million diagnosed each year [3]. ATC is prevalent in older patients, typically over 65 years old, and in females, with a 2:1 female-male ratio [4]. Although ATC is rare, it accounts for about 50% of thyroid cancer-related deaths [5]. Patients with ATC live an average of 3 to 6 months after their diagnosis due to malignancy's rapid onset and ability to metastasize both locally and distally, often in the lungs, bone, and brain [6, 7].

Because of ATC's highly invasive and obscure behavior, treating the disease is a complex undertaking. Standard treatment options for ATC, as with other types of cancer, include excisional surgery, external beam radiation therapy, and chemotherapy, often using taxanes, anthracyclines, and platinum analogs. These treatments are usually administered as a combination of all 3, known as multimodal therapy. The 1-year overall survival (OS) for patients with ATC treated with multimodal therapy remains below 20% and has not changed in the last 20 years. However, in a 20-year retrospective study, it was found that multimodal therapy has an impact on survival with a 1-year and 2-year OS of 59% and 42%, respectively [5]. However, because ATC cells are highly undifferentiated and can resemble noncancerous thyroid cells, detecting and diagnosing the disease proves to be a challenge [8].

An innovative, emerging oncology strategy known as theranostics has shown promise for treating thyroid cancer. Theranostics integrates diagnosis and therapy to target and eradicate cancer. Since cancer presents differently in each patient, theranostics is also used to provide individualized care that follows the unique progression of the malignancy. A widely recognized theranostic for thyroid cancer known as radioactive iodine (RAI) therapy uses radioactive iodine-131 to kill the cancer cells, especially for tumors that have metastasized to other parts of the body. Differentiated thyroid cancer, specifically, responds well to RAI therapy because of the presence of the sodium/iodide symporter (NIS). This transmembrane glycoprotein uptakes iodine necessary for proper thyroid function. Unfortunately, because of the dedifferentiated nature of ATC, the NIS is not expressed on the cells and, therefore, cannot take up the radioactive iodine used for eradication [9, 10].

Although ATC is resistant to RAI therapy, it has proven to be useful for other types of thyroid cancer; we can look for similar mechanisms to administer radiation or cytotoxicity inside the cancer cells. Biomarkers are a key element in theranostics since they are the targets that are detected and manipulated to induce cancer cell damage and death. RAI therapy falls under the category of radioligand therapy as it combines a small molecule with a therapeutic radionuclide to target the cancer cells, specifically biomarkers that are overexpressed in cancer cells but not in normal tissue. The radioligand is then internalized via the designated pathway of the surface marker. Once the radiation is released inside the cell, it induces DNA damage, ultimately leading to cell death [11]. A schematic diagram is shown in Fig. 1 to illustrate the conceptual radioligand therapy using different surface markers.

**Figure 1.**
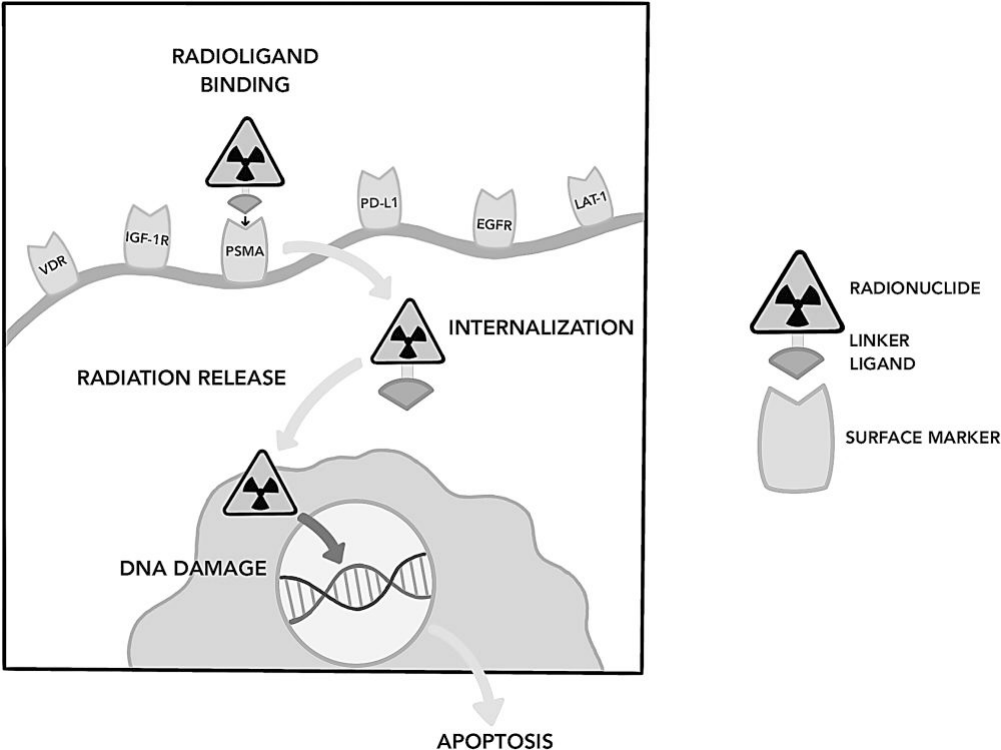
Radioligand therapy mechanism to induce cancer cell death.

Because radioligand therapy, specifically radioiodine therapy, works well for differentiated thyroid cancer but not for ATC, other biomarker targets for theranostics are needed to overcome resistance to treatment in ATC. Previous literature has identified several biomarkers expressed in ATC cells. This review summarizes 6 surface biomarkers found in ATC cells and their potential as targets for future theranostics from the literature search.

## Methodology

We conducted a search of the PubMed database and Google search engine to identify relevant articles for this review. The search was performed using combinations of specific keywords related to the topic of interest, including the names of each surface marker described herein. Titles and abstracts were reviewed to remove irrelevant articles. Then, full texts of potentially relevant articles were evaluated to see if they could be included in the review. We summarized objectives, methods, key findings, and limitations to provide an overview of each surface marker. Our methods are limited to using full-text articles available from PubMed and Google searches; therefore, this review may not encompass studies published in other databases.

## Surface Biomarkers in ATC

Understanding the molecular landscape of ATC cells is crucial for developing theranostic strategies. In this section, we explore 6 surface markers expressed on ATC cells that point to the malignancy's pathogenesis and the potential use of these markers for theranostics and future treatments. Table 1 provides a summary of the biomarkers discussed in this review.

**Table 1.** Surface biomarkers in ATC

Surface biomarker	Location in ATC	Upregulation	Reference(s)
Prostate-specific membrane antigen	Neovasculature	Yes	Rizzo et al [12]
Vitamin D receptor	Tumor cell membrane, nuclear membrane, cytosol	Yes/No	Clinckspoor et al [13] Peng et al [14]
IGF-1 receptor	Tumor cell membrane	Yes	Buck and Mulvihill [15]
Programmed death-ligand 1	Tumor Cell membrane	Yes	Cameselle-Garcia et al [16]
Epidermal growth factor receptor	Tumor Cell membrane	Yes	Schiff et al [17]
L-type amino acid transporter	Tumor Cell membrane	Yes	Enomoto et al [18]

Abbreviations: ATC, anaplastic thyroid cancer.

### Prostate-specific Membrane Antigen Expression in ATC

Prostate-specific membrane antigen (PSMA) is a type II transmembrane glycoprotein receptor with multiple contributions to cellular processes. In a general context, PSMA exhibits folate hydrolase and neuropeptidase activities to break down folate and peptides, respectively [19, 20]. PSMA's folate hydrolase activity contributes to the folate metabolism pathway, which is vital for DNA synthesis and repair, ultimately contributing to cell survival and proliferation—an advantage for cancer cells [21].

#### Location

As the name indicates, PSMA is prevalent in prostate cancer [22]. PSMA has also been detected in other types of malignancies, including breast cancer, lung cancer, colorectal cancer, renal cell cancer, and anaplastic thyroid cancer, where it functions as a neoangiogenesis biomarker, indicating new blood vessel formation in tumors [23, 24]. Although PSMA is confirmed to be on the surface of prostate cancer cells, further studies need to be conducted to determine if the same applies to ATC. As a surface biomarker, it can be internalized through endocytosis via clathrin-coated pits, making it an ideal candidate for theranostics [25].

#### Expression in ATC

PSMA is upregulated in ATC; however, its expression is significantly reduced compared to differentiated and poorly differentiated thyroid cancers. This is likely due to the reduced blood vessel density found in ATC compared to other types of thyroid cancer [12].

#### PSMA in ATC radiotherapy

Because it is highly expressed in prostate cancer, clinicians use PSMA as a target for imaging techniques and therapies such as lutetium-177 prostate-specific membrane antigen radioligand therapy and actinium-225 prostate-specific membrane antigen radioligand therapy to treat prostate cancer. Its success in radioligand therapy may also have implications for treating ATC [22]. In a retrospective study performed by Wächter et al [26]. gallium-68 prostate-specific membrane antigen positron emission tomography/computed tomography (PET/CT) was tested to see if it was more precise than the standard fluorodeoxyglucose F-18 PET/CT for detecting PSMA to track the progression of ATC in a patient and potentially use gallium-68 as a treatment. Unfortunately, there was variation among the patients' results, with the standard imaging technique (fluorodeoxyglucose F-18 PET/CT) superior to gallium-68 prostate-specific membrane antigen PET/CT in detecting the presence of ATC in the patients. However, immunostaining confirmed the presence of PSMA in most of the tumor tissue samples, specifically in the neo vasculature. Further studies will need to be conducted with a large sample size, but because several tissue sections expressed PSMA, this surface marker may be promising for detecting and treating ATC in the future.

### Vitamin D Receptor Expression in ATC

Vitamin D receptor (VDR), when bound to the active form of vitamin D (1α,25-dihydroxyvitamin D3 hormone) on the nuclear membrane, serves as a transcription factor that regulates the expression of genes that influence calcium and phosphate absorption necessary for bone health. Vitamin D and VDR activity also play a crucial role in preventing chronic diseases such as osteoporosis, arteriosclerosis, and diabetes [27]. VDR/vitamin D binding in cancer cells prevents tumor growth and induces apoptosis [28].

#### Location

VDR is found throughout the cell—in the nucleus and cytoplasm, as well as the plasma membrane [29]. Its expression, particularly on the surface of cancer cells, makes it a potential candidate for radioligand therapy and other theranostics. It is found across various tissues, including the kidneys, hair follicles, bone, skin, and stomach, to name a few [30]. VDR has been reported in several cancer types as well, such as breast cancer, colon cancer, and leukemia [28].

#### Expression in ATC

VDR's widespread expression throughout the body reflects its diverse biological functions when bound to vitamin D. However, the expression levels of VDR vary depending on the type of tissue. For example, VDR in normal tissue is sufficient to perform its functions. On the other hand, in tumor tissue, VDR expression is typically downregulated, which can impair the cancer cells' ability to bind to vitamin D and allow them to bypass checkpoints, encouraging cell proliferation and tumor progression [28]. The varying expression levels of VDR are also apparent in ATC tissue, as some report loss of VDR expression is the majority of their samples, but others have confirmed VDR expression in ATC-derived cancer stem cell tissue [13, 14].

#### VDR in vitamin D therapy

Due to its role in inhibiting cancer cell proliferation and promoting apoptosis, VDR can also serve as a potential therapeutic target for various types of cancer, including ATC. Many studies suggest that vitamin D and its analogs exert anticancer properties through VDR. For example, the activation of VDR using calcitriol, the primary active form of vitamin D, has been shown to downregulate cyclin and other cell cycle mediators, resulting in cell cycle arrest at the G0/G1 phase [31]. It can also inhibit angiogenesis and metastasis by downregulating vascular endothelial growth factor and other proangiogenic factors [32, 33].

Although there are variable levels of VDR expression, evidence suggests that VDR agonists may be of therapeutic benefit, especially in more differentiated thyroid cancer types such as papillary thyroid cancer. The function of VDR in more aggressive forms of thyroid cancer, such as ATC, is less clear and needs to be explored further. However, calcitriol-treated ATC cell lines revealed reduced proliferation, pointing to the possible use of VDR agonists in the management of this aggressive cancer [13]. Yet, targeting treatments for VDR in ATC remains limited because this highly differentiated tumor often loses expression of VDR and other differentiation markers. Therefore, there is a need for combination therapies that can potentially restore VDR expression or sensitize the ATC cells to VDR-mediated effects. Combination therapies have been used to overcome these limitations in cancer types with reduced expression of VDR. For instance, calcitriol in combination with chemotherapy or tyrosine kinase inhibitors has been shown, in preclinical trials, to exert anticancer effects such as the induction of apoptosis and inhibition of cell proliferation in a synergistic manner [34]. Ongoing research is focused on optimizing these combination therapies and identifying biomarkers that can predict response to VDR-targeted treatments in ATC.

### IGF-1 Receptor Expression in ATC

IGF-1 receptor (IGF-1R) is a transmembrane receptor tyrosine kinase that contributes to cell growth and development. It interacts with 2 ligands, IGF-1 and IGF-2, which leads to the activation of intracellular signaling cascades that are important for cell proliferation, differentiation, and survival [35]. When IGF-1R is activated, it triggers the PI3kinase-Akt signaling and Ras/Raf/MAPK pathways, promoting cell growth and maturation [36].

#### Location

Overexpression of IGF-1R has been observed in various cancers, such as breast, lung, and prostate cancer, where it is associated with aggressive tumor behavior and resistance to therapy [37]. It is typically found in the cell membrane of tumor cells [38]. Like PSMA, IGF-1R can be internalized through endocytosis, regulated by insulin receptor substrate-1 [39].

#### Expression in ATC

Lawnicka et al [40] analyzed the serum concentrations of IGF-1R in patients with thyroid cancer, including ATC, and found significantly elevated concentrations in the blood samples of patients with ATC compared to the healthy control group. Their results indicate the potential for IGF-R1 to distinguish ATC from other types of thyroid cancer and be a suitable candidate for radioligand therapy to treat ATC. Because of its high expression in ATC and its ability to be internalized, it may be a promising theranostic target for ATC in the future.

#### IGF-1R in ATC treatment

IGF-1R's upregulation in many cancer cells makes it a potential target for several cancer treatments, especially those involving small molecule inhibitors and monoclonal antibodies. In preclinical studies, these therapeutic strategies exhibited inhibiting tumor cell proliferation and induced apoptosis [15, 41]. In the context of thyroid cancer, unfortunately, targeting IGF-1R has not been as successful in clinical trials, with some reporting high toxicity and low efficacy [42]. This suggests that while IGF-1R can be a valid target in ATC cells because of its location, it may work better when combined with other therapeutic agents, such as BRAF or MEK inhibitors, to improve patient outcomes, as demonstrated with other types of cancer, such as melanoma [43].

### Programmed Death-Ligand 1 Expression in ATC

Programmed death-ligand 1 (PD-L1) is a transmembrane protein that interacts with the immune system, specifically in the immune checkpoint process that maintains self-tolerance and limits the risk of autoimmunity in the cell. PD-L1 binds to the PD-1 receptor to inhibit T-cell activation, which triggers an immune response. Tumor cells, however, use this mechanism to avoid the immune checkpoints, avoiding death [44].

#### Location

PD-L1 is typically expressed on the surface of tumor cells. It is also expressed in the tumor microenvironment, specifically on dendritic cells, macrophages, and fibroblasts, which correlates with tumor growth and immune evasion in many cancer types [45]. The location of PD-L1 on the surface of the cells allows it to be internalized by endocytosis using clathrin-coated vesicles, making it a potential delivery agent for targeted therapy [46].

#### Expression in ATC

PD-L1 has also been reported in ATC. In a study performed by Cameselle-Garcia et al [16], samples from 15 ATC cases and 13 poorly differentiated thyroid cancer cases were observed for the expression of PD-L1 and several types of immune cells in the tumor microenvironment. They found that PD-L1 expression was more common in ATC cases (60%) compared to poorly differentiated thyroid cancer cases (7.7%). Its upregulation in ATC contributes to the cells' ability to avoid immune checkpoints, highlighting the aggressive nature of the cancer.

#### PD-L1 clinical trials in ATC treament

PD-L1 is associated with poor prognosis and, therefore, has become a target of many therapies for treating various cancer types such as melanoma, liver cancer, and gastric cancer [45]. Immune checkpoint inhibitors such as pembrolizumab and nivolumab have been investigated for their ability to block the interaction between PD-L1 and PD-1 on T-cells. This therapeutic strategy has enhanced the immune system's ability to attack tumor cells in many cancers [47, 48].

In early clinical trials, PD-1/PD-L1 inhibitors, when combined with targeted therapy, have also shown promise in patients with ATC [49]. A nonrandomized clinical trial aimed to determine whether treatment with matched targeted therapy combined with a PD-L1 inhibitor was associated with improved OS in patients with ATC. This phase 2 clinical trial assigned treatments based on tumor mutation status. Patients with BRAF V600E mutations (cohort 1) received vemurafenib/cobimetinib plus atezolizumab. Patients with RAS (NRAS, KRAS, or HRAS) or NF1/2 mutations (cohort 2) received cobimetinib plus atezolizumab. Patients without BRAF V600E, RAS, or NF1/2 mutations (cohort 3) were treated with bevacizumab plus atezolizumab. For this study, all ATC patients meeting the eligibility criteria were enrolled from 2017 to 2021. The primary outcome of the study was the median OS for each of the targeted therapy cohorts compared with the historical median OS of 5 months. A total of 43 patients with ATC were enrolled, with 42 included in the primary analysis. The median OS of all 3 cohorts was 19 months [95% confidence interval (CI), 7.79-43.24], which was higher than the historical median OS. Cohort 1 demonstrated the longest median OS of 43 months [95% CI, 16–not estimable (NE)] and progression-free survival (PFS) of 13.9 months (95% CI, 6.6-64.1). Cohort 2 had a median OS of 8.7 months (95% CI, 5.1-37.0) and a PFS of 4.8 months (95% CI, 1.8-14.7). Cohort 3, treated with a vascular endothelial growth factor inhibitor, achieved a median OS of 6.21 months (95% CI, 4.1-NE) and a PFS of 1.3 months (95% CI, 1.3-NE). The outcome of cohort 1 demonstrates the potential of this approach in patients carrying a BRAF V600E mutation [50]. There is a variable response of thyroid cancers to PD-L1 inhibition because of genetic mutations present in ATC, creating the need for further research focused on identifying biomarkers that may predict which patients will benefit from this type of therapy.

### Epidermal Growth Factor Receptor Expression in ATC

Epidermal growth factor receptor (EGFR) is a transmembrane protein that contributes to several cellular processes, including growth and survival. As a part of the receptor tyrosine kinase family, it binds to epidermal growth factor (EGF), followed by its dimerization. This activates the PI3K/AKT and Ras/Raf/MAPK pathways in the cell [51]. Like the other surface biomarkers, EGFR is also internalized by the cell through clathrin-mediated endocytosis, making it a potential candidate for theranostics [52].

#### Location

EGFR is expressed throughout the body, specifically in epithelial tissue involved in the repair mechanism. It is also expressed in many different types of cancer, including lung cancer, breast cancer, colorectal cancer, and glioblastoma. It is important to note that although EGFR is found on the surface of cancer cells, its presence in normal tissue such as the skin, lungs, and kidneys creates a challenge for targeting the protein to treat cancer. However, it is still studied as a crucial target for therapy. Bispecific monoclonal antibodies, for instance, can deliver cytotoxic drugs into the cancer cells by binding to the target tumor antigen without affecting normal tissue [53, 54]. Therefore, EGFR can still be explored as a potential candidate for targeted therapy to treat ATC.

#### Expression in ATC

EGFR is consistently overexpressed in ATC cells, as determined by Schiff et al [17] in both human ATC tissue (in vivo) and ATC cell lines (in vitro).

#### EGFR as a therapy target in ATC

EGFR inhibitors such as gefitinib and erlotinib have shown promise in treating cancers that overexpress EGFR or express mutated EGFR [55]. The study performed by Schiff et al [17] tested the effect of gefitinib on ATC cell lines and nude mice, where they observed that the drug blocked EGFR binding to EGF, effectively inhibiting tumor cell proliferation and inducing apoptosis. Their results encouraged further exploration into the effect of EGFR inhibitors in human clinical trials. Unfortunately, because of the aggressive and variable nature of ATC, some patients with ATC do not respond well to this particular therapy [56]. However, there are clinical trials currently underway that are evaluating the effect of EGFR inhibitors administered with other targeted therapies or immunotherapies to improve patient outcomes, specifically with lung cancer [57]. Further research will need to be conducted to determine if similar therapies can be applied to ATC.

### L-type Amino Acid Transporter 1 Expression in ATC

L-type amino acid transporter 1 (LAT-1) is a surface protein involved in transporting large neutral amino acids and thyroid hormones across the cell membrane, aiding in protein synthesis and cellular metabolism. It is also crucial for transporting nutrients across the blood-brain barrier to supplement the central nervous system [58]. LAT-1 is a part of the L-amino acid transport system, which works to regulate the transport of amino acids into and out of the cells. It is sodium-independent, meaning that the movement of amino acids is influenced by their concentration gradients instead of sodium's concentration gradient [59]. Although LAT-1 typically transports drugs into the cell to induce therapeutic effects, it can also be internalized by ubiquitylation-mediated endocytosis, opening a new avenue for this protein to be used in theranostics [60].

#### Location

LAT-1 is normally expressed in the plasma membrane of cells found in several areas of the body including the brain, smooth muscle, bladder, and bone marrow [61]. It is also upregulated in several types of cancer such as lung cancer, pancreatic cancer, prostate cancer, breast cancer, and thyroid cancer [62].

#### Expression in ATC

There is limited knowledge about the specific role of LAT-1 in ATC. However, 1 study found that LAT-1 is highly expressed on the surface of ATC tumor cells and hardly detected in surrounding thyroid tissue, making it a potential target for therapy [18].

#### LAT-1 inhibitors in ATC treatment

In tumors, LAT-1 aids in the transport of nutrients needed for growth and cell proliferation. Because of this trait, it has become a promising therapeutic target to eradicate cancer cells [63]. Monoclonal antibodies and small molecule inhibitors designed to target LAT-1 have the potential to disrupt amino acid transport and cell growth [64, 65]. In ATC specifically, previous literature for LAT-1 therapies is limited. However, JPH203, a LAT-1 inhibitor, has shown promise for reducing tumor growth in ATC cell lines and patient-derived xenograft models [18]. LAT-1 is also a key player in boron neutron capture therapy (BNCT), a targeted therapy that utilizes boronated compounds to shrink tumors [66]. BNCT has not yet been studied in ATC; however, because LAT-1 in present in ATC cells, BNCT may be considered for patients in the future. This treatment is further discussed in the next section.

## Future Use of LAT-1 in BNCT for ATC Treatment

As scientists strive to increase the overall survival of patients with ATC through the expansion of effective diagnostic and treatment modalities, BNCT as a theranostic optimistically offers comprehensive diagnosis and precise termination within a single tool. In BNCT, tumor-specific ligands containing boron-10—an isotopic element both rare and inert in living cells—are injected into patients' blood vessels. Due to its designed specificity, high relative quantities of the BNCT ligand will be retained inside targeted tumor cells. Subsequently, patients undergo local, low-energy, thermal neutron radiation, which will exclusively react with the boron-10 contained in target cells. A capture reaction occurs in which the boron-10 in the ligands and the emitted low-energy thermal neutrons react to yield boron-11, lithium-7, and alpha particles. The high energy and short path length of the alpha particles directly disrupt the target cells' DNA while containing cellular damage within 5 to 9 µm of the cells, thus yielding the precise termination of tumor cells [67].

Furthermore, BNCT has been modified for theranostics by tagging ligands with a radiotracer. This modification allows scientists to initially diagnose tumor cells through imaging and identifying localized cells containing high amounts of the radio-traced BNCT ligands. When tumor cells are identified, low-thermal neutron radiation can be promptly applied to terminate diagnosed cells. Thus, both the diagnosis and treatment of tumor cells can be accomplished through the single administration of radio-traced BNCT ligand.

One of the earliest applications of BNCT was the targeted termination of malignant melanoma. Malignant melanoma is an aggressive skin cancer that is both radioresistant and treatment resistant. Mishma and Yoshino previously proposed p-boronophenylalanine (BPA) as an effective tumor-specific ligand due to its structural similarity to tyrosine, an amino acid precursor to melanin [68]. While further studies are necessary before clinical application, a preclinical trial applied BNCT on 8 patients (6 patients with complete presentation and 2 patients with partial presentation of malignant melanoma), reporting full recovery in all patients except 1 who remained with partial presentation [69]. As for theranostics, scientists have also designed radio-traced BPA. By tagging BPA with fluorine-18 isotopes, scientists were able to detect and diagnose concentrated localization of BPA using PET scans before applying neutron radiation to terminate the malignant melanoma [70].

Toward employing BNCT as a theranostic for ATC, previously discovered ATC biomarkers with high specificity could direct the design of radio-traced BNCT ligands. Designing a ligand with high specificity for ATC, low toxicity, and prompt clearance posttreatment can reduce unintended damage to healthy cells during therapy [71]. Recent studies have identified ATC-specific transporter proteins that point to a promising application for BNCT theranostics. Enomoto et al [18] identified LAT-1 to be sensitively expressed in human ATC vs noncancerous thyroidal tissue. Future studies could investigate LAT-1-specific ligands as a framework for designing structural analogs as candidate ligands for BNCT theranostic approaches to treat ATC.

## Restoring NIS Expression for ATC Therapy

In addition to targeting the surface markers described earlier for theranostics, another potential therapeutic strategy is to reintroduce the NIS in ATC cells to allow for the administration of RAI therapy. As mentioned previously, the NIS is necessary for the uptake of iodine for normal thyroid function and is leveraged for RAI therapy to deliver radioactive iodine into the thyroid cancer cells. ATC has not responded well to RAI therapy because its dedifferentiated nature suppresses the expression of NIS [9, 10].

To combat ATC's inability to concentrate iodide, recent efforts have investigated the potential of restoring NIS expression in ATC to help patients respond positively to RAI therapy. One study by Schmohl et al [72] aimed to optimize NIS-mediated radionuclide imaging and therapy in ATC cell lines by delivering a plasmid expressing NIS through EGFR-specific nanoparticle vectors (LPEI-PEG-GE11/NIS). Results showed a successful transfection and high specificity in the cell lines, correlating with iodide uptake and EGFR expression levels. In their in vivo experiments, systemic polyplex (nanoparticle vector) administration resulted in significant tumor-specific radioiodine accumulation in ATC xenograft mouse models, with higher uptake in SW1736 tumors compared to Hth74 tumors (both tumor types are from ATC cell lines). Following radioiodine accumulation, tumor growth was reduced substantially, prolonging survival and showing the potential of this novel gene delivery system for ATC therapy.

Nanoparticles were also utilized in a study by Li et al [73], specifically lipid peptide-mRNA nanoparticles to deliver NIS mRNA into ATC cells. NIS expression was enhanced over 10-fold in ATC cell lines. In vivo studies involving ATC tumor-bearing mice injected with the nanoparticles showed a significant increase in NIS expression and radioiodine uptake, reducing tumor growth. The researchers of this study suggest the potential of these nanoparticles as adjuvant therapy for radioiodine-resistant thyroid tumors such as ATC to improve the efficacy of the treatment.

Another study by Xu et al [74] found that capsaicin, a transient receptor potential cation channel subfamily V member agonist, induces differentiation in ATC cells by upregulating thyroid transcription factors and iodine-metabolizing genes and promoting NIS glycosylation and membrane trafficking to enhance radioiodine uptake, offering a new therapeutic strategy for patients with ATC.

Heydarzadeh et al [75] explored the potential of a phytomedicine called Rutin to target “flip-flop”—a phenomenon in which iodide and glucose uptake alternate in thyroid cancer—in ATC (SW1736) cells. Using MTT assays, spectrophotometric assays, colorimetric assays, and quantitative RT-PCR, it was found that Rutin reduced cell viability, increased NIS and iodide uptake, and decreased GLUT1/GLUT3 expression. This study highlights Rutin's potential as a therapeutic agent by restoring cell differentiation and NIS expression for increased iodine uptake and inhibiting glucose metabolism.

These studies, along with a few others, demonstrate the potential of novel strategies leveraging the standard RAI therapy used for other forms of thyroid cancer with the hope that the same can be achieved in ATC tumors.

## Conclusion

Identifying surface biomarkers offers a promising path for advancing theranostics and other targeted therapies for ATC, a highly aggressive and lethal form of thyroid cancer. Given that traditional radioiodine therapy is largely ineffective in patients with ATC due to the cancer's acquired inability to uptake iodine, alternative treatment strategies that focus on specific surface markers present a crucial opportunity. These biomarkers provide unique molecular targets that can be exploited for precision therapies, enabling the development of treatments that are more selective and less toxic than conventional therapies. By targeting specific surface biomarkers, innovative therapeutic approaches such as BNCT, gene therapies, monoclonal antibodies, antibody-drug conjugates, and radioligand therapies can be explored. BNCT, for example, offers a novel method where boron-containing compounds are delivered selectively to cancer cells. Upon neutron irradiation, the boron atoms release localized high-energy radiation that destroys cancer cells while sparing healthy tissues. Restoring NIS expression is another potential strategy for treating patients with ATC, as its role in RAI uptake is crucial for successfully treating other forms of thyroid cancer.

Such targeted therapies hold great potential for improving outcomes in patients with ATC, where current treatment options are limited and survival rates remain dismal. Further research into surface biomarkers could also lead to earlier detection of ATC, as these markers may serve as diagnostic tools, allowing clinicians to identify the cancer at a more treatable stage. This would be particularly significant for cancer like ATC, where early intervention could make a substantial difference in prognosis. Additionally, targeting these biomarkers could prevent or mitigate metastasis, a common and deadly feature of ATC, by interrupting critical pathways involved in tumor growth and spread.

The biomarkers discussed in this review all have similarities that make them ideal for targeted therapies such as radioligand therapy, as they are expressed on the surface of tumor cells and are able to internalize and deliver therapeutic agents into the cancer cells. They can be targeted specifically to avoid damaging normal surrounding tissue. Further studies need to be performed to better understand the latter criterion in ATC tumors and develop therapeutic agents specific to these surface markers expressed on ATC cells.

While all these surface markers have the potential to be leveraged for theranostics, successfully restoring NIS expression in patients may be a significant stride in eradicating ATC tumors. The high uptake of radioiodine and positive response to RAI therapy in differentiated types of thyroid cancer may indicate the potential of ATC responding in the same way. In addition, if LAT-1 can successfully internalize boronated drugs into ATC cells, BNCT is another possible therapy for treating patients with the disease. Further extensive research is needed to form a comprehensive understanding of each surface marker and its roles in ATC tumors to create novel, safe, and effective strategies to improve patient outcomes.

Ultimately, advancing our understanding of surface biomarkers in ATC could pave the way for personalized medicine strategies, where treatments are tailored based on the molecular profile of the patient's tumor. This precision-based approach could significantly improve prognosis, reduce treatment-related toxicity, and increase OS rates for patients with ATC, offering hope in an area of oncology where few effective treatments currently exist.

## Data Availability

Original data generated and analyzed during this study are included in this published article or in the data repositories listed in References.

## Abbreviations

ATC: anaplastic thyroid carcinoma
BNCT: boron neutron capture therapy
BPA: p-boronophenylalanine
EGF: epidermal growth factor
EGFR: epidermal growth factor receptor
IGF-1R: IGF-1 receptor
LAT-1: L-type amino acid transporter 1
NIS: sodium/iodide symporter
OS: overall survival
PD-L1: programmed death-ligand 1
PET/CT: positron emission tomography/computed tomography
PFS: progression-free survival
PSMA: prostate-specific membrane antigen
RAI: radioactive iodine
VDR: vitamin D receptor
